# The Potential Association between Obstructive Sleep Apnea and Diabetic Retinopathy in Severe Obesity—The Role of Hypoxemia

**DOI:** 10.1371/journal.pone.0079521

**Published:** 2013-11-18

**Authors:** Dev Banerjee, Wen Bun Leong, Teresa Arora, Melissa Nolen, Vikas Punamiya, Ron Grunstein, Shahrad Taheri

**Affiliations:** 1 Academic Department of Sleep and Ventilation, Birmingham Heartlands Hospital, Heart of England NHS Foundation Trust, Birmingham, United Kingdom; 2 St Vincent’s Hospital and Woolcock Institute of Medical Research, University of Sydney, Sydney, Australia; 3 Birmingham and Black Country NIHR CLAHRC, University of Birmingham, Birmingham, United Kingdom; 4 Specialist Weight Management Services, Heart of England NHS Foundation Trust, Birmingham, United Kingdom; 5 NHMRC Centre for Integrated Research and Understanding of Sleep (CIRUS) Woolcock Institute of Medical Research, University of Sydney and St Alfred’s Hospital, Sydney, New South Wales, Australia; Azienda Policlinico S. Orsola-Malpighi, Italy

## Abstract

**Background:**

Obstructive sleep apnea (OSA) is common in obese patients with type 2 diabetes mellitus (DM) and may contribute to diabetic microvascular complications.

**Methods:**

To investigate the association between OSA, hypoxemia during sleep, and diabetic retinal complications in severe obesity. This was a prospective observational study of 93 obese patients mean (SD) age: 52(10) years; mean (SD) body mass index (BMI): 47.3(8.3) kg/m^2^) with DM undergoing retinal screening and respiratory monitoring during sleep. OSA was defined as apnea-hypopnea index (AHI) of ≥15 events/hour, resulting in two groups (OSA+ vs. OSA−).

**Results:**

Forty-six patients were OSA+: median (95% CI) AHI = 37(23–74)/hour and 47 were OSA–ve (AHI = 7(4–11)/hour). Both groups were similar for ethnicity, BMI, cardiovascular co-morbidities, diabetes duration, HbA_1c_, and insulin treatment (p>0.05). The OSA+ group was significantly more hypoxemic. There was no significant difference between OSA+ and OSA− groups for the presence of retinopathy (39% vs. 38%). More OSA+ subjects had maculopathy (22% vs. 13%), but this did not reach statistical significance. Logistic regression analyses showed that AHI was not significantly associated with the presence of retinopathy or maculopathy (p>0.05). Whilst minimum oxygen saturation was not significantly associated with retinopathy, it was an independent predictor for the presence of maculopathy OR = 0.79 (95% CI: 0.65–0.95; p<0.05), after adjustment.

**Conclusions:**

The presence of OSA, as determined by AHI, was not associated with diabetic retinal complications. In contrast, severity of hypoxemia during sleep (minimum oxygen saturations) may be an important factor. The importance of hypoxia in the development of retinal complications in patients with OSA remains unclear and further studies assessing the pathogenesis of hypoxemia in patients with OSA and diabetic retinal disease are warranted.

## Introduction

Obstructive sleep apnea (OSA) is characterized by frequent partial or complete cessation of breathing during sleep leading to intermittent low oxygen levels in the blood (hypoxemia) with subsequent low oxygen levels in organs and tissues (hypoxia) [1]. OSA is common, affecting an estimated one in five middle-aged men [2]. The prevalence of OSA rises with increasing adiposity [2] and it is estimated that up to 75% of those with a body mass index (BMI) above 40 kg/m^2^ (extreme obesity) have OSA [3]. With the increasing prevalence of obesity, sleep clinics are more frequently serving severely obese patients with OSA and diabetes mellitus. OSA has been reported to be associated with insulin resistance, metabolic syndrome, type 2 diabetes mellitus (DM) and vascular disease [4]–[7]. Moderate to severe OSA has been associated with an increased risk of DM [8]. The reported prevalence of OSA in patients with DM ranges from 23 to 86% [7], [9].

Frequent episodes of hypoxemia during sleep in OSA have been reported to be associated with raised inflammatory mediators [10]. In DM, inflammatory mediators are also elevated [11], and are contributory to diabetic micro-vascular complications such as diabetic retinal disease (diabetic retinopathy (DR) and diabetic maculopathy (DMac)), a leading cause of blindness [12]. Some studies have shown that concomitant OSA in DM patients might be associated with significantly more DR cases, and more advanced sight-threatening DMac, with the suggestion that OSA is an independent risk factor for diabetic retinal complications [13], [14]. These studies used oxygenation as part of their definition of OSA, suggesting that the intermittent hypoxemia and re-oxygenation episodes in OSA lead to a greater oxidative stress and inflammatory responses, and hence worsening of diabetic retinal disease [15]. There is very little information, however, regarding the association between sleep-disordered breathing as defined by apnea-hypopnea frequency and diabetic retinal complications, or whether other hypoxemia parameters are important, particularly the degree of hypoxemia during sleep (e.g. minimum oxygen saturations or time spent under 90% oxygen saturations). Furthermore, little has been studied in patients with severe obesity, a high-risk group for OSA and DM. We hypothesized that DM patients with severe obesity and moderate-to-severe OSA (as defined by apnea – hypopnea frequency) will have a higher prevalence of diabetic retinal disease compared to those without OSA. We therefore undertook a cross-sectional evaluation of a cohort of consecutive severe obese patients with DM undergoing assessment with routine clinical retinal screening and overnight respiratory sleep monitoring.

## Materials and Methods

### Study Design and Study Subjects

A cohort of consecutive patients attending the regional specialist weight management service at Birmingham Heartlands Hospital, UK between 2009 and 2011 was studied. Routine clinical data collected included age, gender, self-reported ethnicity, weight, BMI, cardiovascular co-morbidities (hypertension and coronary artery disease), duration of DM, DM medications, HbA_1c_ (DCCT-aligned and IFCC), and renal function (creatinine and estimated glomerular filtration rate (eGFR)).

### Respiratory Sleep Monitoring Methods

Patients underwent home portable respiratory monitoring during sleep using the Embletta™ (Embla systems). The protocol for this system is used as part of standard clinical care in the current service setting. All patients were shown how to utilize the equipment by a trained physiologist in sleep medicine on the afternoon of the test night. Measurement channels included airflow using a nasal pressure device, chest and abdominal movements by inductance plethysmography, oxygen saturation through pulse oximetry, heart rate, and body position. The staff observed the patient undergoing a trial of self-application before leaving the sleep physiology department. Devices were returned the next day and studies were downloaded and reviewed for data quality. Patient filled in a sleep diary for the night of time to bed and time to estimated sleep and time wake up to assess the matching with the objective sleep respiratory signals. Studies showing at least four hours of good quality respiratory signals were regarded as acceptable. If any data were deemed inadequate, the patient was offered a retest. All data were manually scored by blinded sleep trained physiologists and then rescored by author DB for confirmation. Author DB was blinded to any diabetic eye screening data. All parameters were scored based on respiratory event during sleep scoring guidelines published by the American Academy of Sleep Medicine [16]. Respiratory parameter data collected included: apnea/hypopnea index (AHI), mean and minimum oxygen saturation during sleep, and the percentage of time spent under 90% oxygen saturation while asleep. OSA in this study was defined as the presence of an AHI greater or equal (≥)15/hour (OSA+). Those with AHI of less than 15/hour were regarded as the OSA– group. The AHI was used as the diagnostic parameter for OSA and not the oxygen desaturation index (4%) or ODI. ODI was not measured in this study. The cut-off point for AHI of 15/hour was selected as it forms part of the clinical decision making determining whether or not to treat with continuous positive airway pressure (CPAP).

### Retinal Screening Methods

DR data were obtained from the routine local screening program based on the English National Screening Program for Diabetic retinopathy (ENSPDR). Two 45° fields per eye (1 fovea-centered, 1 disc-centered) are obtained on an annual basis. ENSPDR has developed guidance on the classification of the severity of diabetic retinopathy in England and Wales [17]. All diabetes patients attending the clinic underwent annual retinal screening and data were acquired from the most recent screening assessment. Presence or absence of DR and DMac were recorded. DMac is defined as any evidence of exudate, microaneurysm or haemorrhage with 1 disc diameter of the center of fovea [17].

### Statistical Analysis

All statistical analyses were carried out using the Statistical Package for Social Sciences, version 19 (SPSS, Chicago, IL). Data analysis was performed on anonymized patients as part of service evaluation, therefore no formal ethical approval was required as per recommendation from the UK National Research Ethics Service [18]. Normally distributed data are reported as mean ± standard deviation (SD) and analyzed using an independent t-test whilst skewed data are reported as median and interquartile range (IQR) and the Mann Whitney U-test was used for analysis. Categorical data were analyzed using the chi-squared test. Univariate analyses were carried out to examine factors associated with diabetic retinal disease. Logistic regression analyses were conducted and three models constructed to examine associations between 1) AHI (continuous); 2) mean oxygen saturation; 3) minimum oxygen saturation (min O_2_); and 4) % time spent under 90% oxygen saturation; and DR as well as DMac. Model 1 was unadjusted, Model 2 adjusted for age, gender and ethnicity, and Model 3 was further adjusted for DM duration, insulin treatment and HbA_1c_, hypertension, and coronary artery disease. Results were reported as odds ratio (OR) with 95% confidence interval (95% CI). A p value of <0.05 was considered statistically significant.

## Results

There were a total of 121 DM patients eligible, but three patients’ sleep respiratory data were not analyzable, and 26 patients did not have the complete available retinopathy screening data; therefore, a total of 93 eligible patients were included in the study. No patient had a diagnosis of central sleep apnea.

The mean (SD) age of the patients was 52(10) years and 59% of the patients were females. The majority of patients were of white European origin (69%) and 23% were of South Asian origin. Mean (SD) weight and BMI were 130.4(25.3) Kg and 47.3(8.3) Kg/m^2^, respectively. Sixty-three per cent of patients suffered from hypertension. Median (IQR) duration for DM was 6(2–10) years with a median (IQR) HbA_1c_ of 62(51–77) mmol/mol or 7.8(6.8–9.2)%. The majority of patients were on diabetes medications (81.7%) and 33% of the patients were on insulin treatment. Median (IQR) AHI was 14(7–36) events/hour with a median (IQR) minimum oxygen saturation 82(74–85)% and median (IQR) % time spent under 90% oxygen saturation of 3(1–12)% for the total sample.

Table 1 shows the patient characteristics, according to the presence or absence of OSA, determined by AHI. Of the 93 patients, 46(51%) had an AHI≥15 events/hour with a median (IQR) AHI of 37(23–74)/hour (OSA+), compared to those with an AHI <15/hour; median (IQR) AHI = 7(4–11)/hour (OSA−). Both OSA+ and OSA− groups were well matched for ethnicity, weight, BMI, co-morbidities, diabetes duration, HbA1c and DM medication (p>0.05). The OSA+ group were slightly older than those without OSA (54(10) vs. 49(10) years, p = 0.01) with a greater proportion of males (66% vs. 25%, p = 0.002).

**Table 1:** Sample characteristics of 93 patients according to presence/absence of obstructive sleep apnea.

	OSA−(n = 47)	OSA+(n = 46)	p value
Age (years)	49±10	54±10	0.010
Gender, n (%)			0.002
Female, 55 (59)	35 (75)	20 (43.5)	
Ethnicity, n (%)			0.716
White European	34 (72)	30 (65)	
South Asian	9 (19)	12 (26)	
Afro-Carribean	4 (9)	4 (9)	
Weight (kg)	127±23	135±27	0.142
BMI (kg/m^2^)	47.1±8.1	47.6±8.5	0.776
Systolic BP (mmHg)	139±15	146±23	0.177
Diastolic BP (mmHg)	83±10	85±12	0.446
Co-morbidities, n (%)			
Hypertension	26 (55)	33 (72)	0.100
CAD	5 (11)	7 (15)	0.510
Diabetes parameters			
DM duration (years)	6 (3–11)	6 (2–10)	0.423
DM medications, n (%)			0.795
None	9 (19)	8 (17)	
One	10 (21)	14 (30)	
Two	20 (43)	17 (37)	
Three or more	8 (17)	7 (15)	
HbA_1c_ (%)	7.7 (6.7–9.3)	8.1 (7.1–9.2)	0.267
Insulin treatment	12 (26)	19 (41)	0.123
Respiratory parameters			
AHI (/hour)	7 (4–11)	37 (23–74)	<0.001
Mean O2 saturation (%)	94 (92–95)	93 (91–94)	0.001
Minimum O2 saturation (%)	84 (82–88)	76 (71–82)	<0.001
% Time spent <90% saturation	1 (0–3)	11 (2–25)	<0.001
Renal parameters			
Creatinine	67 (57–79)	78 (66–93)	0.002
eGFR (ml/min/1.73 m^2^)	90 (77–90)	83 (61–90)	0.068
Retinopathy outcome, n (%)			
Retinopathy present	18 (38)	18 (39)	0.934
Maculopathy present	6 (13)	10 (22)	0.252

Data are presented as mean±standard deviation, number (%), or median (interquartile range).

OSA = obstructive sleep apnea; BMI = body mass index; CAD = coronary artery disease; DM = diabetes mellitus; BP = blood pressure; eGFR = estimated glomerular filtration rate.

p values were calculated using either independent t-test, chi square or Mann Whitney U-test.

Table 2 shows the retinal screening results. The overall prevalence of DR and DMac were present in 38.7% and 17.2%, respectively. No significant differences were found between the presence/absence of OSA for DR (39% vs. 38%, p = 0.765). However more OSA+ patients had DMac compared to OSA− patients (22% vs. 13%), but this did not reach statistical significance (p = 0.252).

**Table 2:** Diabetic retinopathy in 93 patients according to presence/absence of obstructive sleep apnea.

	OSA− (n = 47) n (%)	OSA+ (n = 46) n (%)	p value
**Both eyes**			
No retinopathy	29 (61.7)	28 (60.9)	0.765
Background retinopathy	15 (31.9)	13 (28.3)	
Pre-proliferative retinopathy	1 (2.1)	3 (6.5)	
Proliferative retinopathy	2 (4.3)	2 (4.3)	
Maculopathy	6 (12.8)	10 (21.7)	0.252
Photocoagulation	3 (6.4)	2 (4.3)	0.664
**Right eye**			
No retinopathy	32 (68.1)	30 (65.2)	0.943
Background retinopathy	12 (25.5)	12 (26.1)	
Pre-proliferative retinopathy	2 (4.3)	2 (4.3)	
Proliferative retinopathy	1 (2.1)	2 (4.3)	
Maculopathy	5 (10.6)	5 (10.9)	0.971
Photocoagulation	3 (6.4)	2 (4.3)	0.664
**Left eye**			
No retinopathy	33 (70.2)	31 (67.4)	0.777
Background retinopathy	11 (23.4)	10 (21.7)	
Pre-proliferative retinopathy	1 (2.1)	3 (6.5)	
Proliferative retinopathy	2 (4.3)	2 (4.3)	
Maculopathy	5 (10.6)	10 (21.7)	0.146
Photocoagulation	3 (6.4)	2 (4.3)	0.664

OSA = obstructive sleep apnea.

p values derived from chi square test.

Univariate logistic regression identified factors significantly associated with diabetic retinal disease. Diabetic retinal disease was significantly associated with diabetes duration (OR 1.12; 95% CI: 1.04–1.21) and insulin treatment (OR 8.21; 95% CI: 3.08–21.83). DMac was associated with HbA_1c_ (OR1.48; 95% CI: 1.06–2.06), DM duration (OR 1.16; 95% CI: 1.06–1.26), insulin treatment (OR24.29; 95% CI: 5.02–117.57) and hypertension (OR4.98; 95% CI: 1.06–23.44). The results of our multivariate logistic regression analyses are shown in Table 3. We observed, after adjustment, no relationship between DR and AHI, mean oxygen saturation, minimum oxygen saturation, and % time spent <90% oxygen saturation. After adjustment, the presence of DMac was not associated with AHI, mean oxygen saturation, and % time spent <90% oxygen saturation. Minimum oxygen saturation was, however, after adjustment, an independent and significant predictor for the presence of DMac OR = 0.79 (95% CI: 0.65–0.95; p<0.05)).

**Table 3:** Logistic regression analyses assessing the presence of diabetic retinopathy (DR) and maculopathy (DMac) with four respiratory parameters.

Presence of retinopathy (DR)	Model 1	Model 2	Model 3
AHI	1.01 (0.99–1.02)	1.01 (0.99–1.02)	1.00 (0.98–1.02)
Mean oxygen saturation	0.89 (0.77–1.03)	0.90 (0.77–1.05)	0.83 (0.67–1.02)
Minimum oxygen saturation	0.95* (0.90–1.00)	0.95 (0.89–1.00)	0.93 (0.86–1.01)
Time spent <90% oxygen saturation	1.03 (1.00–1.05)	1.03 (1.00–1.06)	1.03 (1.00–1.06)
**Presence of maculopathy (DMac)**	**Model 1**	**Model 2**	**Model 3**
AHI	1.01 (1.00–1.03)	1.01 (0.99–1.03)	1.01 (0.98–1.04)
Mean oxygen saturation	0.93 (0.79–1.08)	0.92 (0.77–1.09)	0.80 (0.59–1.07)
Minimum oxygen saturation	0.91** (0.85–0.97)	0.90** (0.84–0.97)	0.79* (0.65–0.95)
% Time spent <90% oxygen saturation	1.02 (0.99–1.04)	1.02 (0.99–1.04)	1.03 (0.99–1.08)

Model 1: unadjusted.

Model 2: adjusted for age, gender, ethnicity.

Model 3: further adjusted for diabetes mellitus duration, insulin treatment, HbA_1c_, hypertension, and coronary artery disease.

* p<0.05;

** p<0.01.

## Discussion

Retinal disease is a well-recognized micro-vascular complication of DM [19]. To our knowledge, this is the first DR prevalence study on extreme obese individuals. Our study shows that the overall prevalence of DR amongst extreme obese DM patients was 38.7%. This is slightly higher than the reported global DR prevalence of 34.6% despite our patients being 6 years younger and having shorter DM duration (6.0 vs. 7.9 years) compared to the study by Yau et al [20]. A study on the National Health and Nutrition Examination Survey study from 2005 to 2008 reported an even lower estimated DR prevalence of 28% [21]. The discrepancy may be due to the fact that our study only has severely obese DM patients, who are an increasingly important at-risk group.

Our study did not show any significant difference in the prevalence of diabetic retinal disease between those who had moderate to severe OSA compared to those who did not. We examined the relationship between diabetic retinal disease and respiratory parameters using logistic regression analysis. After correction for age, gender, ethnicity, diabetes duration, usage of insulin and the presence of hypertension, there was no association found between mean oxygen saturations, and % time spent under 90% oxygen saturations and retinal disease. Our results were consistent with the Japanese studies, which did not find any correlation with the above respiratory parameters [14], [15].

There has been much interest in the role of OSA in the development of micro-vascular complications in patients with DM; this study showed that OSA, when measured by using the apnea – hypopnea index, has no association with retinal complications. Only one study investigated the effect of AHI and DR and it was performed in 98 veterans who are predominantly male. Although this study found a significant positive correlation between DR and AHI, the result was not adjusted for potential confounders [22]. The patients were also much older (9 years older) with more severe OSA (mean AHI 31 events/hr) compared to our study [22]. Other studies have found a positive association between ODI and DR [13], [14]. Shiba and colleagues compared 116 proliferative (PDR) and 48 non-proliferative DR (NPDR) inpatients [14]. They reported a 43% correlation between ODI and PDR [14]. Our study was not designed to investigate PDR and OSA as we only have 4 episodes of PDR in our study. The other study from Oxford investigated 118 males and found significant correlation between ODI and HbA_1c_ with DR [13]. However, this study is not comparable to ours as it used ODI rather than AHI and the patients had a much longer duration of DM (10 years) and were a much older group (67 years) compared to our study [13].

Logistic regression analyses showed that the degree of hypoxemia, determined by minimum oxygen saturation during sleep, was associated with the development of DMac, but not DR. As the OSA group was more hypoxemic than the group without moderate to severe OSA, this may explain why the OSA+ had a higher prevalence of DMac. The relationship between OSA and diabetic retinal disease is important given the risk of blindness [19]. West et al, in a study of 118 DM male patients, reported that OSA (mean (SD) ODI 20.9(16.6) per hour vs. 2.8(2.1) per hour in the control group) was associated with DMac (r^2^ = 0.3) [13]. In another study by Shiba et al of 219 DM patients, it was found that, after adjusting for potential confounders, the lowest oxygen saturation and not ODI or % time spent under 90% oxygen saturation significantly correlated with the development of proliferative DR (OR = 0.93, 95% CI0.88–0.99; p = 0.02) [15]. It is of note that the diagnosis of OSA for both the studies was made on the basis of oxygenation (ODI) and not breathing parameters (AHI) that the severity of obesity as measured by BMI was significantly lower than our study.

The findings of our study show that minimum oxygen saturation may be associated with DMac supports the findings by Shiba et al [15]. Although our study measured AHI rather than ODI, the combination of results from our and Shiba et al. [14] studies on oxygenation and diabetic eye micro-vascular disease, may indicate that intermittent hypoxemia and lowest oxygen saturation (and not apnea/hypopnea frequency) is independently associated with diabetic retinal disease especially DMac. The animal [23] and human [24] studies have shown that chronic intermittent hypoxia is linked to a reduction in endothelial relaxation and greater vasoconstriction. This may impair oxygenation to the retina leading to diabetic retinal disease, particularly in vulnerable areas such as the macula. Apart from dysfunction in vaso-reactivity, oxidative stress and systemic inflammation may also play a part. The study by Yamauchi et al showed that ODI was correlated to oxidative stress markers in subjects with OSA [25]. Vascular endothelial growth factor (VEGF) has been found to be elevated in hypoxic OSA patients [10] and may be associated with the neovascularization process in ischemic retinal disease [11]. Further longitudinal studies are required to examine the causal effect of hypoxia from OSA on diabetic retinal disease.

Apart from intermittent hypoxia, the other potential mechanisms for greater DMac in OSA patients may be due to elevated BP and severity of DM. Hypertension is strongly associated with OSA [5], [26]. Although not statistically significant, systolic BP for our OSA+ patients was higher compared to OSA− (146 vs. 139 mmHg). It is well-recognized that BP plays an important role in DR progression as shown in the UKPDS study [27]. Insulin use is sometimes used as a surrogate marker for greater severity of DM and has been found to have a significant impact on DR in overweight T2D patients [28]. In our study, a greater proportion in OSA+ group was treated with insulin (41% vs. 26%) compared to OSA− group. This is unsurprising as there is an association between greater insulin resistance and impaired glucose metabolism with severity of OSA [4], [8]. HbA_1c_ level was also 0.4% higher in the OSA+ group and this is also associated with severe DR [29].

Our study also examined creatinine and eGFR levels. There was a significant higher creatinine level and a non-significant lower eGFR in OSA+ group. These findings may act as a reassurance in the probable greater early development of diabetic retinal disease as part of DM micro-vascular complications in our OSA+ group. The impact of correcting OSA on micro-vascular complications remains unknown but a study recently by Mason et al looking at the impact of CPAP on patients with OSA (defined as ODI>10 events/hr or AHI>15 per hour) on macular edema showed no improvement in the disease, but interestingly, some improvement in visual acuity [30].

Our study benefits from well-matched groups that are representative of the severely obese population and the ethnic diversity of our local population. The OSA+ and OSA− groups were well matched for BMI, co-morbidities, diabetes duration, and diabetes medications. The OSA+ group had more men and was older than the OSA− but these differences were accounted for when undergoing statistical analyses. Also, in our analyses, we observed the expected and previously reported factors associated with diabetic retinal disease. The limitations of our study include its cross-sectional design and lack of information regarding other potentially important factors such as smoking. Visual acuity data were also not available.

### Conclusions

This study of severely obese patients with DM found no association between OSA and DR but there was a higher prevalence of DMac in those with moderate to severe OSA. Further analysis suggests that the severity of hypoxemia, particularly minimum oxygen saturation has an independent association with the development of maculopathy but the exact mechanism for this remains unclear. As this OSA group was more hypoxemic, it is hypothesized that hypoxemia and not apnea/hypopnea frequency per se is the key factor in developing diabetic micro-vascular complications. The other likely potential mechanisms were greater systemic hypertension and severity of DM in OSA. The impact of CPAP on micro-vascular retinal complications in OSA patients remains undetermined, but further RCTs may be warranted to specifically look at the hypoxemic OSA group.
